# Identification and Characterization of *Botryosphaeria dothidea* Associated with Sweet Cherry (*Prunus avium* L.) Branch Dieback Disease in Greenhouses of Liaoning, China

**DOI:** 10.3390/biology15020183

**Published:** 2026-01-19

**Authors:** Qidong Dai, Qijing Zhang, Yao Chen, Feng Cai, Mingli He, Jiayin Ai

**Affiliations:** Liaoning Institute of Pomology, Yingkou 115009, China; daiqd@126.com (Q.D.); cheny9020@163.com (Y.C.); caifengln@hotmail.com (F.C.); minglihehe@hotmail.com (M.H.); jiayinai@hotmail.com (J.A.)

**Keywords:** sweet cherry, *Prunus avium*, *Botryosphaeria dothidea*, branch dieback, molecular characterization, pathogenicity assay, phylogenetic analysis

## Abstract

Between 2022 and 2024, a severe branch dieback disease was observed affecting over 6% of sweet cherry trees of the ‘Tieton’ cultivar in commercial greenhouses in southern Liaoning Province, China. Symptoms primarily occurred at the top of young branches. At the early stage of disease onset, the lesions appeared as dark brown, irregularly shaped areas with a moist surface; as the disease progressed, these lesions turned dry and rotten, leading to tree decline symptoms in sweet cherry trees. Disease diagnosis was carried out in sweet cherry greenhouses across Liaoning Province, where 24 diseased samples were collected and 14 fungal isolates were obtained therefrom. Based on morphological traits, cultural characteristics, and multi-locus phylogenetic analyses of the internal transcribed spacer (ITS) region, beta-tubulin (*TUB2*) gene, and translation elongation factor 1-α (*TEF1*) gene, these isolates were identified as *Botryosphaeria dothidea*. Two representative isolates, namely zdcy-1 and zdcy-2, were selected for pathogenicity assays. Both mycelial plug and spore suspension inoculation methods confirmed the pathogenicity of the pathogen. The biological characteristic assays revealed that the optimal temperature range for the pathogen’s mycelial growth on PDA medium was 25–28 °C, and the optimal pH range was 6.0–8.0. This study improves the understanding of branch dieback disease in sweet cherry orchards in China, enriches the knowledge regarding the geographical distribution, host range, and infection sites of the pathogen, and provides novel insights for the management of sweet cherry diseases.

## Simple Summary

China is one of the major sweet cherry-producing countries worldwide, and in particular, its greenhouse cultivation area accounts for a substantial proportion globally. Scientific management of sweet cherry diseases is a critical issue to be addressed in the greenhouse sweet cherry cultivation industry. Identifying the species and occurrence regularity of pathogenic microorganisms is the foundation of scientific disease prevention and control. The results of this study indicate that the branch dieback disease of the sweet cherry cultivar ‘Tieton’ is a fungal disease caused by the infection of *Botryosphaeria dothidea*. Notably, when the host plants are subjected to environmental stress, this pathogen can transform from an endophyte into a pathogenic fungus, aggravating disease occurrence and even triggering disease prevalence. This disease not only impairs the tree vigor of sweet cherries but also degrades fruit quality and reduces yield. In view of the differences in the biological adaptability of the pathogen, optimizing greenhouse cultivation conditions, rationally planting resistant cultivars, and applying chemical fungicides in a timely manner are of great significance for controlling the prevalence of this disease.

## 1. Introduction

Sweet cherries (*Prunus avium* L.) belong to the Rosaceae family and are widely cultivated in countries with temperate climates. In recent years, driven by its high nutritional and commercial value, the sweet cherry industry has experienced rapid development in China. At present, the total cultivation area of sweet cherries in China has reached 230 kha, among which the greenhouse cultivation area accounts for 20 kha, and the total output amounts to 1.8 million tons [1,2]. However, severe yield losses have been caused by pathogen infection. *Alternaria* post-harvest decay, *Colletotrichum* leaf spot, and *Diaporthe* sp. trunk canker are the major diseases in sweet cherry production [3,4,5].

*Botryosphaeria dothidea* is one of the most important endophytic or latent pathogenic fungi, infecting a broad range of host plants in forestry and horticulture [6,7,8]. This pathogen exhibits pathogenicity toward *Pinus* spp., *Photinia* spp., *Eucalyptus* spp., *Populus* spp., and *Jatropha* spp. [9,10,11,12,13], and also colonizes economically important crops in agricultural ecosystems—particularly *Pyrus* spp., *Malus* spp., *Prunus* spp., *Vaccinium* spp.—as well as the ornamental plant *Osmanthus* spp. [14,15,16].

The primary symptoms induced by this pathogen include stem canker, branch dieback, kino exudation from the trunk, leaf lesions, fruit rot, and postharvest fruit decay [17,18,19,20]; in severe cases, it can even lead to the death of host plants [21]. *B. dothidea* is reported to cause canker and dieback in *Eucalyptus* spp. [22,23], leaf blotch in *Salix babylonica* [12], stem canker in apple (*Malus* spp.) [24,25], branch and fruit ring rot in apple and pear [26,27,28], canker and dieback in grapevines [29,30], branch dieback in olive [31], bud and shoot blight in pistachio [32,33,34,35], brown rot in walnut [36], shoot blight in peach [37], canker in almond [21,38], stem blight in blueberry [39], and gummosis in peach trees [40,41].

The genus *Botryosphaeria* is generally regarded as a species complex. Owing to the high level of intraspecific variation among *Botryosphaeria* spp. isolates, which overlaps with that of other related species, morphological characteristics are no longer routinely employed for the identification of *Botryosphaeria* spp. [8]. It is recommended that sequence data of the internal transcribed spacer (ITS) region, combined with those of the translation elongation factor 1-α (*TEF1*) and beta-tubulin (*TUB2*) genes, can effectively distinguish closely related species [42].

*Botryosphaeria dothidea* is one of the important pathogens distributed worldwide, which can typically induce symptoms including dieback, canker, and gummosis [15]. Fungi belonging to the family Botryosphaeriaceae are common dieback-causing pathogens of *Prunus* species (including plum, peach, and apricot) in South Africa, and are also associated with canker diseases of almond in the United States [15,21]. In Spain, *B. dothidea* can cause branch dieback of olive, while in the United States, it can induce pistachio shoot blight disease [31,32]. In China, researchers have reported that this pathogen can cause branch canker in Japanese cherry (*Prunus serrulata*) [43], as well as leaf spot and gummosis in sweet cherry (*Prunus avium* L.) [44,45]. However, there have been no reports of *B. dothidea* inducing branch dieback in sweet cherry trees to date.

Branch dieback diseases are highly destructive and can significantly reduce the yield and shorten the lifespan of cherry orchards. These diseases can infect the tender branches of sweet cherry trees; in severe cases, they may even lead to the death of trees shortly after orchard establishment. The authors collected branch dieback disease samples from sweet cherry trees in commercial orchards located in southern Liaoning Province, China. The pathogens were isolated and identified, and the association between *B. dothidea* and branch dieback disease was investigated by integrating analyses of morphological characteristics, pathogenicity assays, and phylogenetic data. This study aimed to characterize the fungal pathogens responsible for branch dieback disease in sweet cherry trees in Liaoning Province, northern China.

## 2. Materials and Methods

### 2.1. Collection of Disease Samples and Morphological Identification of the Pathogens

A survey was conducted on 5-year-old sweet cherry trees in greenhouses located in Dalian City (39.27° N, 121.79° E) and Yingkou City (40.21° N, 122.12° E), Liaoning Province, from 2022 to 2024. Branch dieback disease was observed on the sweet cherry cultivar ‘Tieton’, with the disease incidence exceeding 6% of the surveyed trees. To identify the causal agent of this dieback disease, 24 diseased samples were collected from commercial sweet cherry orchards, from which 14 fungal isolates were obtained. Tissue segments (4 mm × 4 mm) were excised from the lesion margins of the symptomatic branches. The infected tissues were surface-sterilized by immersion in 1% sodium hypochlorite solution for 20 s, followed by 75% ethanol solution for 10 s. Subsequently, the tissues were rinsed with sterile distilled water and transferred onto potato dextrose agar (PDA) medium (composed of 12% potato extract, 1.6% glucose, and 1.6% agar). To induce sporulation, the isolates were cultured on oat-agar medium at 25 °C under ultraviolet light, where pycnidia and conidia were produced. After 7 days of incubation, 2 mm mycelial plugs were cut from the colony margins and transferred to fresh PDA medium. This purification procedure was repeated three times to obtain pure cultures [46].

Colony morphological characteristics were examined following the criteria described by Rayner [47] after 7 days of incubation on PDA at 25 °C. For each isolate, the length and width of 30 conidia were measured, and the mean values were calculated. Digital images of the morphological features were captured using a digital camera (Model Nikon 100, Nikon Corporation, Tokyo, Japan), and conidial dimensions were determined using Phmias 2008 ver 3.0 software (Phoenix Optical Co., Ltd., Shangrao, China).

### 2.2. The Effects of Temperature and pH Values on B. dothideaMycelial Growth

Temperature Assay: sterile 5-mm-diameter cork borers were used to excise mycelial plugs from the colony margins of the isolates. The plugs were then inoculated onto PDA medium and incubated in the dark at 5, 10, 15, 20, 25, 28, 30, and 35 °C, respectively. Following 7 days of incubation, the longitudinal and transverse diameters of the colonies were measured. Mean values and standard errors were subsequently calculated, and the assay was performed in triplicate.

pH Assay: sterilized PDA medium was cooled to 50 °C, and its pH was adjusted to gradients of 4.0, 5.0, 6.0, 7.0, 8.0, 9.0, and 10.0 using 0.1 mol/L hydrochloric acid and 0.1 mol/L sodium hydroxide. pH values were verified with a digital pH meter (Model PHS-25, Yidian Scientific Instruments Co., Ltd., Shanghai, China). Mycelial plugs were inoculated onto PDA media with different pH gradients and incubated in the dark at 25 °C for 7 days. Colony diameter measurement and data statistical analysis were performed using the same methods as described for the temperature assay.

### 2.3. DNA Extraction, Amplification, and Sequencing of the Isolates

Purified *B. dothidea* isolates preserved in 50% glycerol at −20 °C were cultured on PDA plates at 25 °C for 5 days. Genomic DNA was extracted from the representative isolates zdcy-1, zdcy-2, zdcy-3, and zdcy-4 using a modified CTAB method [48]. The rDNA-ITS region was amplified with primers ITS1 and ITS4 [49]; a partial fragment of the *TUB2* gene was amplified with primers Bt2a and Bt2b [50]; and a partial fragment of the *TEF1* gene was amplified with primers EF1-728F and EF1-986R [51] via polymerase chain reaction (PCR) (Table 1). Template DNA was dissolved in 50 μL of sterile water. The PCR mixture had a final volume of 25 μL, containing 1.5 units of Taq DNA polymerase, 1× buffer-MgCl_2_ mixture (10 mM Tris-HCl, 1.0 mM MgCl_2_, 40 mM KCl), 0.15 mM of each dNTP, and 0.2 μM of each primer, with the volume made up using sterile water. The PCR amplification program was as follows: initial denaturation at 94 °C for 2 min; 40 cycles of denaturation at 94 °C for 30 s, annealing at gene-specific temperatures (55 °C for rDNA-ITS, 53 °C for *TEF1*, and 58 °C for *TUB2*) for 45 s, and extension at 72 °C for 1 min; and a final extension at 72 °C for 5 min. PCR products were separated by electrophoresis on 1.5% agarose gels, stained with ethidium bromide, and visualized under ultraviolet light. Electrophoretic band sizes were estimated using standard molecular weight markers. PCR products were purified using a PCR purification kit (Omega Biotek, Norcross, GA, USA). Primer synthesis and PCR product sequencing were performed by Sangon Biotech Co., Ltd. (Shanghai, China).

### 2.4. Phylogenetic Analyses of B. dothidea Isolates

Sequences of *Botryosphaeria* spp. corresponding to the rDNA-ITS, *TEF1*, and *TUB2* regions were retrieved from the NCBI GenBank database. The homologous sequences of these three loci from isolates zdcy-1, zdcy-2, zdcy-3, and zdcy-4 were analyzed using DNAMAN v. 9.0 software (Lynnon Biosoft, San Ramon, CA, USA), after which the sequences were submitted to GenBank to obtain accession numbers. Sequence homology comparisons were performed using the NCBI BLASTn programme, and reference sequences of *Botryosphaeria* spp. were downloaded from the GenBank database. The ITS, *TEF1*, and *TUB2* sequences were aligned with MEGA 7.0 software, followed by sequence trimming and concatenation using PhyloSuite v3.0 software. A phylogenetic tree was constructed via the neighbor-joining (NJ) algorithm [52], with node support validated by the bootstrap method based on 1000 replicate datasets and a confidence threshold set at 50%. The concatenated sequences of the four isolates in this study were compared with those of five reference *B. dothidea* isolates (CMW 8000, CMW 7780, CMW 7999, CMW 9075, and CBS 100564). Additionally, 15 sequences representing closely related *Botryosphaeria* species (*B. ribis*, *B. parva*, *B. fusispora*, *B. obtusa*, *B. rhodina*, *B. corticis*, *B. lutea*, and *B. stevensii*) were included in the phylogenetic analysis. *Guignardiaphiloprina* CMW 7063 was designated as the outgroup for tree rooting (Table 2).

### 2.5. Pathogenicity Assays of B. dothidea Isolates

Spore Suspension Inoculation: In June 2024, one-year-old shoot branches of the ‘Tieton’ cultivar were collected from commercial greenhouses in the Dalian region. Thirty excised branches were inoculated with the representative strain zdcy-1 to verify pathogenicity. Prior to inoculation, the branches were cut into 30-cm-long segments. The branches were first rinsed with tap water to remove impurities, surface-sterilized by immersion in 75% ethanol for 30 s followed by 1% sodium hypochlorite for 10 s, and then rinsed thoroughly with sterile water. The disinfected branches were air-dried in a laminar flow hood. Sterile filter paper was soaked in a conidial suspension (10^6^ conidia/mL) supplemented with 0.1% Tween-20, placed on the wound sites, and secured with plastic cling wrap. For the control group, branches were treated with sterile filter paper soaked in sterile water and wrapped in the same manner.

Mycelial Plug Inoculation: Mycelial plugs of the isolate zdcy-2 were punched from the margins of 7-day-old colonies grown on PDA medium and inoculated onto excised one-year-old sweet cherry shoot segments. The mycelial plugs were inserted into sterilized wounds on the branches, which were created using a cork borer. A piece of sterile cotton soaked in sterile distilled water was placed over each mycelial plug, and the inoculation sites were sealed tightly with parafilm. Control branches were inoculated with sterile PDA plugs without mycelial growth.

Both inoculation experiments were conducted with three replicates, and the treated branches were incubated at 25 °C under a 12-h photoperiod and 70% relative humidity. Seven days post-inoculation, the mean lesion length (cm) was measured and subjected to analysis of variance (ANOVA). The disease incidence for the spore suspension and mycelial plug inoculation treatments was calculated separately as follows: the number of symptomatic shoots divided by the total number of inoculated shoots in the experiment. Pathogens were re-isolated from the symptomatic branches; colonies morphologically consistent with *B. dothidea* were subjected to morphological and molecular identification to satisfy Koch’s postulates.

### 2.6. Statistical Analysis

The mean values and standard errors were calculated using Microsoft Excel 2007 software (Microsoft Corporation, Redmond, WA, USA), and one-way analysis of variance (ANOVA) was performed using DPS 7.05 software (Ruifeng Information Technology Co., Ltd., Hangzhou, China). A *p*-value < 0.05 was considered statistically significant. Graphs were plotted using Origin 2021 software (OriginLab Corporation, Northampton, MA, USA). All data were expressed as the mean ± standard error, with vertical lines representing the standard errors.

## 3. Results

### 3.1. Identification and Characterization of B. dothidea

**Figure 1:**
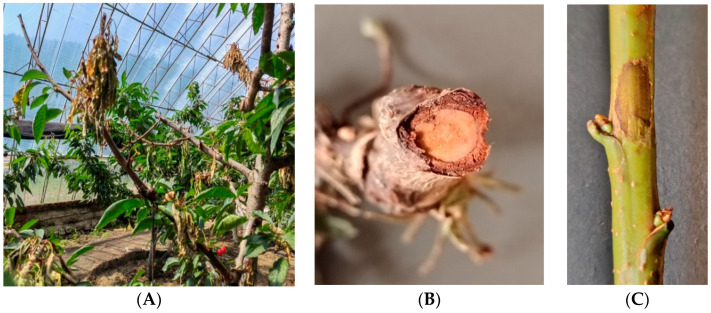
Typical symptoms of *B. dothidea* infection associated with branch dieback in field-grown sweet cherry trees. (**A**) Withered and necrotic apical branches; (**B**) Cross-section of a diseased sweet cherry branch; (**C**) Early-stage symptoms on young shoots.

*Botryosphaeria dothidea* infected the main and lateral branches of sweet cherry trees, causing branch dieback and rot. This disease was prone to occur on the apices of young shoots. At the early stage of infection, the affected branches developed brown, fusiform lesions with irregular shapes and moist surfaces; as the disease progressed, these lesions desiccated and rotted, gradually expanding to invade the phloem tissue and eventually leading to the wilting and death of branches and leaves (Figure 1A–C).

**Figure 2:**
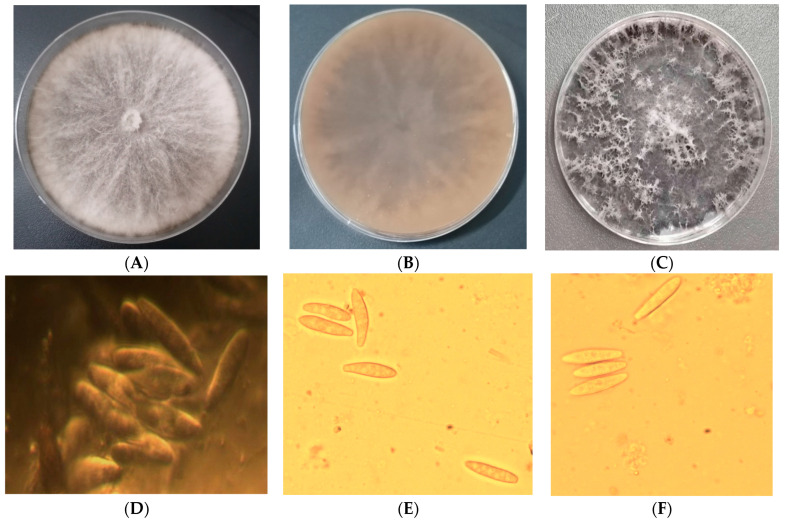
Morphological characteristics for identification of *B. dothidea* isolated from sweet cherry cultivar ‘Tieton’ in China. (**A**,**B**) Front and reverse views of colonies cultured on PDA medium for 7 days; (**C**) Colony morphology following 14 days of incubation on PDA medium; (**D**) Conidial discharge from pycnidia of *B. dothidea*; (**E**) Conidia of *B. dothidea* strain zdcy-1; (**F**) Conidia of *B. dothidea* strain zdcy-2. Scale bar: (**D**–**F**) = 10 μm.

When cultured on PDA medium, the colonies initially appeared olivaceous, ranging from sparse to moderately dense, with occasional aerial hyphae extending to the lid of the culture dish. The colony margins were smooth to serrated; as the colonies matured, they turned dark gray or black (Figure 2A–C). Abundant conidia were produced on oat agar medium. The conidia were narrowly fusiform, with bluntly rounded bases, unicellular, hyaline, smooth-walled, and contained granular inclusions. They measured 19.6–25.3 × 4.2–6.2 µm (*n* = 30), with an average size of 22.6 × 4.9 µm and length/width ratios ranging from 3.3 to 6.1. The pycnidia formed on oat agar medium were black and spherical, with dimensions of 54.6–86.4 × 56.2–86.3 µm (*n* = 30) (Figure 2D–F). The morphological characteristics of the colonies and conidia were consistent with the descriptions of *B. dothidea* [42,53].

### 3.2. The Effect of Temperature on B. dothidea Mycelial Growth

**Figure 3:**
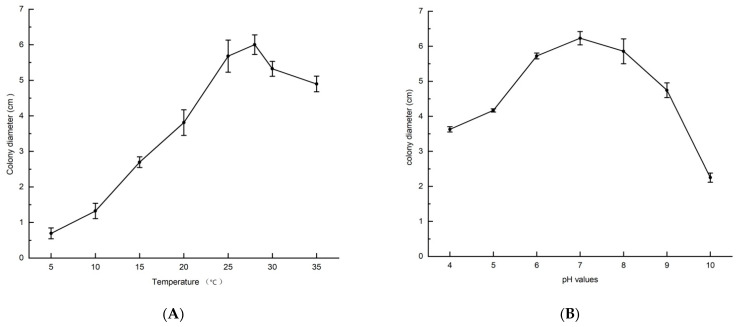
Biological characteristics of *B. dothidea* cultured on PDA medium. (**A**) Effects of temperature on mycelial growth; (**B**) Effects of pH values on mycelial growth. Error bars indicate the standard deviation (*n* = 3).

The pathogens were cultured on PDA medium at 25 °C in the dark for 7 days across a gradient of temperatures. The results showed that the isolates were able to grow at temperatures ranging from 5 °C to 35 °C. The optimal temperature range for mycelial growth was 25–28 °C, with the colony diameter reaching 5.68 ± 0.45 cm at 25 °C and 6.00 ± 0.27 cm at 28 °C. At temperatures between 5 °C and 10 °C, mycelial growth of the isolates was significantly inhibited, with the colony diameters measuring only 0.69 ± 0.15 cm at 5 °C and 1.32 ± 0.22 cm at 10 °C. Overall, the pathogen exhibited stronger adaptability to high temperatures than to low temperatures (Figure 3A).

### 3.3. The Effect of pH Values on B. dothidea Mycelial Growth

The isolates were capable of growing on PDA medium across a pH range of 4.0–10.0. The optimal pH range for mycelial growth of the isolates was 6.0–8.0, with colony diameters reaching 5.72 ± 0.09 cm, 6.23 ± 0.19 cm, and 5.86 ± 0.36 cm at pH 6.0, 7.0, and 8.0, respectively. In contrast, mycelial growth was significantly inhibited at pH 10.0, with the colony diameter measuring only 2.25 ± 0.13 cm (Figure 3B).

### 3.4. Phylogenetic Analyses of B. dothidea

Based on the concatenated sequence datasets of the internal transcribed spacer (ITS-rDNA), translation elongation factor 1-α (*TEF1*), and β-tubulin (*TUB2*) regions, the *Botryosphaeria* spp. analyzed in this study were clustered into two major clades. One clade comprised four isolates from this study (zdcy-1, zdcy-2, zdcy-3, and zdcy-4), five reference *B. dothidea* isolates (CMW 8000, CMW 7780, CMW 7999, CMW 9075, and CBS 100564), and five closely related *Botryosphaeria* species (*B. fusispora*, *B. obtusa*, *B. rhodina*, *B. corticis*, and *B. stevensii*). Within this clade, zdcy-1, zdcy-2, zdcy-3, and zdcy-4 were grouped together with the five reference *B. dothidea* isolates. The other clade consisted of three closely related *Botryosphaeria* species (*B. ribis*, *B. parva*, and *B. lutea*).

**Figure 4:**
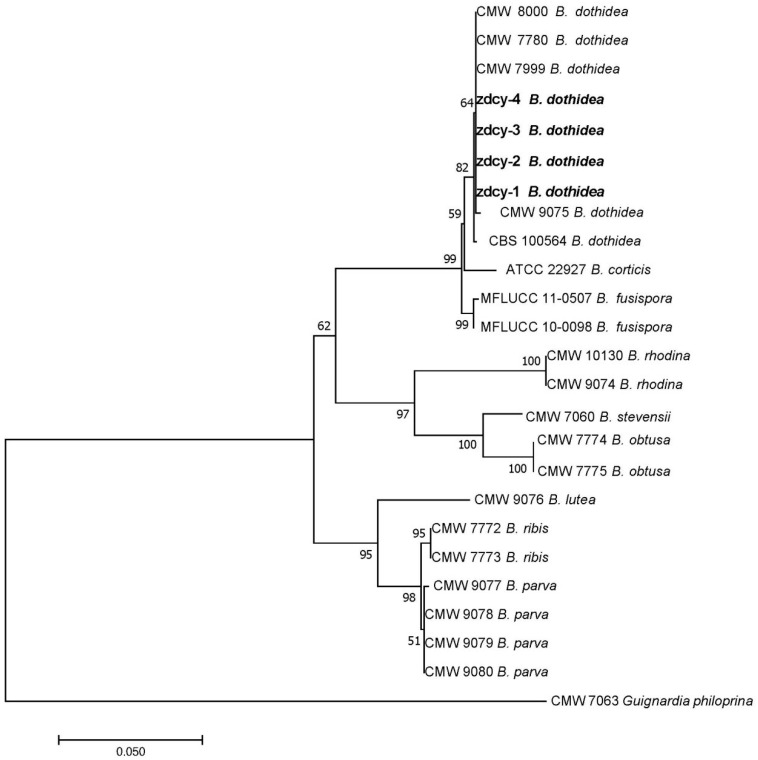
Phylogenetic tree constructed by neighbor-joining analysis based on concatenated sequences of the rDNA internal transcribed spacer (ITS), translation elongation factor 1-α (*TEF1*), and β-tubulin (*TUB2*) genes. The analysis included four *B. dothidea* isolates (zdcy-1, zdcy-2, zdcy-3, and zdcy-4), with *Guignardiaphiloprina* CMW 7063 designated as the outgroup. Isolates obtained in the present study are shown in bold. Bootstrap values (>50%) derived from 1000 replicates are displayed at the tree nodes. The scale bar indicates 0.05 nucleotide substitutions per site.

These four isolates from the present study were clustered in the same clade as the reference *B. dothidea* isolates, whereas they exhibited a relatively distant genetic relationship with the other eight closely related *Botryosphaeria* species (*B. fusispora*, *B. obtusa*, *B. rhodina*, *B. corticis*, *B. stevensii*, *B. ribis*, *B. parva*, and *B. lutea*) (Figure 4).

### 3.5. Pathogenicity of B. dothidea

**Figure 5:**
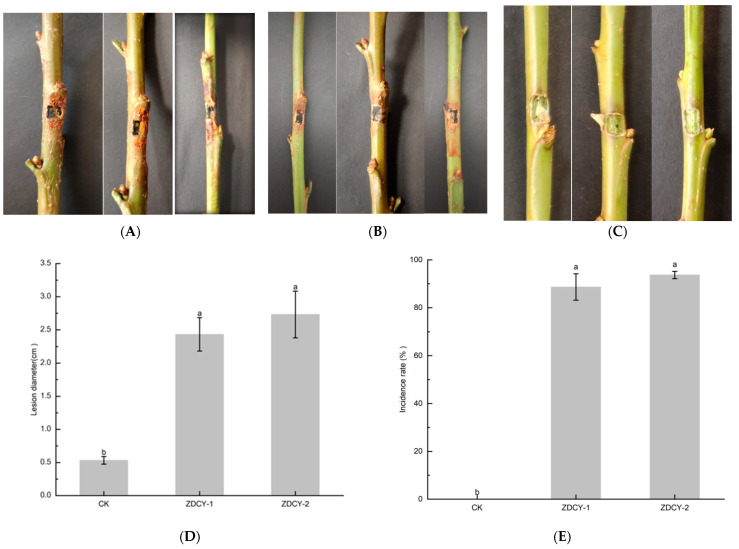
Pathogenicity of *B. dothidea* isolates to *Prunus avium* cv. ‘Tieton’. (**A**) Spores suspension inoculated (zdcy-1) on sweet cherry shoots 7 days post inoculation (7 dpi); (**B**) Mycelium plug (zdcy-2) inoculated on sweet cherry shoots (7 dpi); (**C**) Control group; (**D**) Mean lesion lengths on shoots inoculated by *B. dothidea* isolates (bars indicates the standard errors; letters a and b are used to indicate the significance of differences between different groups (*p* < 0.05); (**E**) Disease incidences of shoots inoculated by *B. dothidea* isolates.

At 7 days post-inoculation on wounded, excised one-year-old shoots of the ‘Tieton’ cultivar, both mycelial plug and spore suspension inoculation assays induced browning and necrosis in all shoots inoculated with *B. dothidea*; the resulting lesions were consistent with those observed under field conditions. In contrast, no symptoms were observed on the control shoots (Figure 5A–C). The mean lesion lengths of shoots inoculated with the spore suspension and mycelial plugs were 2.43 ± 0.25 cm and 2.73 ± 0.35 cm, respectively, which were significantly greater than those of the control groups treated with sterile water or sterile uncolonized PDA plugs. The disease incidence rate reached 88.67% for the spore suspension inoculation and 93.67% for the mycelial plug inoculation (Figure 5D,E). Fungi re-isolated from the symptomatic shoots exhibited morphological characteristics identical to those of the originally inoculated isolates; this identity was further confirmed by ITS-rDNA region sequencing. The re-isolated strains showed 100% sequence homology with the reference *B. dothidea* isolates.

## 4. Discussion

Species belonging to the family Botryosphaeriaceae are regarded as stress-associated pathogens, and any potential environmental changes may render previously uninfected host plants susceptible to infection. Adverse environmental conditions can also induce physiological stress in host plants, thereby creating opportunities for latent pathogens to switch to a pathogenic state [54]. The pathogenic significance of *B. dothidea* has long been underestimated, primarily due to its endophytic lifestyle. In the high-humidity environment of greenhouses, this fungus can shift from an endophytic to a pathogenic phase, which may lead to severe damage to infected host plants.

Conidia of *B. dothidea*, like those of other *Botryosphaeria* spp., are thought to be dispersed over relatively short distances via wind and rain [8]. Disease occurrence is typically associated with abiotic stresses, including drought, physical damage, frost, and suboptimal growing environments. It is hypothesized that *B. dothidea* can infect its host plants through either wounds or natural openings [32]. In recent years, the mechanisms underlying pathogen penetration and evasion of host defense responses have been elucidated [55,56,57,58,59]. Morphological variation in *Botryosphaeria* spp. can manifest in the size, aggregation pattern, and implantation mode of pycnidia across different tissues of a single host plant or among distinct host species. The teleomorph of *B. dothidea* is rarely observed in artificial cultures, whereas the anamorph is ubiquitous [42]. Consistent with this, the sexual morph of *B. dothidea* was not detected in the present study, while its asexual morph was readily identified.

The isolates used for phylogenetic analyses in this study were derived from 13 host genera and 8 different countries spanning six continents. Researchers [60] have reported that in California, *Calosphaeriapulchella*, *Cytosporasorbicola*, and *Eutypalata* are the primary pathogens causing branch dieback disease in sweet cherries. The present study confirms that *B. dothidea* is also a causal agent of this disease in northern China. Additionally, this pathogen can infect sweet cherry leaves andfruits [20]. Thus, we infer that the population size of the pathogen carried by sweet cherry branches and leaves during the growing season is correlated with the incidence of postharvest fruit diseases.

*Botryosphaeria dothidea*, *B. ribis* and *B. parva* are generally recognized as comprising a species complex. *B. dothidea* and *B. ribis* were once regarded as synonymous [61], but current insights from genetic and morphological studies indicate that they are distinct species. ITS or RAPD markers cannot effectively discriminate between these two species [62], whereas Inter-Simple Sequence Repeat (ISSR) markers enable their clear differentiation [63]. In the present study, these species were assigned to separate clades. *Botryosphaeria dothidea*—which corresponds to the anamorph genus *Fusicoccumaesculi*, characterized by hyaline conidia [62]—was clustered in one clade, while *B. ribis*—the anamorph of which belongs to *Diplodia* and produces dark conidia [42]—was grouped in another clade. The asexual stage of *B. dothidea* is often referred to as *Fusicoccumaesculi*, and the anamorph of *B. ribis* is now defined as *Fusicoccumribis*.

This study confirmed that *B. dothidea*, *B. ribis* and *B. parva* are distinct taxa. *Botryosphaeria ribis* and *B. parva* are more closely related species; their anamorphs, which produce dark, ellipsoidal conidia and belong to *Diplodia* [63,64], were clustered in the same clade but formed separate subclades. In contrast, these two species were grouped in a different clade from *B. dothidea*, whose anamorph produces *Fusicoccum*-like conidia [65]. This conclusion is consistent with the findings reported by Zhou [63].

*Botryosphaeria dothidea* can infect stone fruits, and it is particularly known to induce gummosis in peach and cherry trees [40,44]. Inappropriate pruning practices, frost damage, microbial community imbalance [66], and pathogen infection are key factors contributing to the occurrence of gummosis. Based on field symptom observations and in vitro pathogenicity assay results, the present study confirmed that *B. dothidea* is capable of causing branch dieback disease in the ‘Tieton’ sweet cherry cultivar. By contrast, the cultivars ‘Summit’, ‘Hanxiang’, and ‘American Red’—which are widely cultivated in greenhouses across Liaoning Province—exhibit relatively low incidence of this dieback disease.

Branch dieback disease occurred with greater severity under extreme weather conditions (high humidity and elevated temperatures), which imposed excessive stress on the greenhouse-grown sweet cherries surveyed in Liaoning Province. The findings of this study indicated that the optimal temperature range for pathogen growth was 25–28 °C, consistent with the research conclusions reported by Liu [67]. The optimal pH for pathogen growth varied across different host species, and alkaline conditions were unfavorable for pathogen proliferation [68]. The authors recommend the following primary strategies for controlling sweet cherry branch dieback disease in greenhouses: maintaining temperature and humidity within appropriate ranges, promptly removing diseased branches, avoiding mechanical wounds during pruning, and implementing chemical protection at the early stage of disease onset. Additionally, enhancing tree vigor and planting disease-resistant cultivars are also optimal management approaches.

Plant branch diseases caused by *B. dothidea* usually occur as mixed infectionswith other pathogens [60]. Under unfavorable environmental conditions, infection symptoms may become evident, thereby increasing the difficulty of disease prevention and control. Accurate identification of this pathogen is of great significance for understanding, preventing, and managing the disease [69]. The results of this study are consistent with the hypothesis that *B. dothidea* can switch from an endophytic to a pathogenic phase under adverse conditions. This finding highlights the need for local growers to pay close attention to this disease and may also lay a foundation for future diagnostic and control strategies for the disease in China. To develop sustainable plant protection strategies, further in-depth research is required to investigate the effects of resistant cultivars [70], agronomic practices, and novel active ingredients [71] on sweet cherry branch dieback disease.

## 5. Conclusions

This study conducted a systematic investigation into the branch dieback disease that has inflicted severe damage on protected-cultivated sweet cherries in Liaoning Province, northern China, in recent years. Results demonstrated that this disease is an infectious disorder caused by *B. dothidea* infection, and the pathogen exhibits extensive biological adaptability.

The major cultivated cultivar ‘Tieton’ was found to be particularly susceptible to the disease, with marked differences in disease resistance observed among different sweet cherry cultivars. Sunscald, low-temperature stress, and mechanical damage were identified as the primary inducing factors, whereas the high-temperature and high-humidity environment in greenhouses could accelerate the spread and prevalence of the disease.

Maintaining greenhouse temperatures below 22 °C, conducting timely ventilation to reduce humidity, preventing shoots from suffering sunscald and freezing injury, pruning diseased branches appropriately, and spraying alkaline chemical fungicides at the initial stage of disease onset can effectively inhibit pathogen reproduction and reduce the risk of disease outbreaks.

## Figures and Tables

**Table 1:** Primer pairs for the identification of *B. dothidea* associated with sweet cherry branch dieback disease in China.

Gene (Locus)	Primer	Primer Sequence (5′-3′)
rDNA-ITS	ITS1	TCCGTAGGTGAACCTGCGG
	ITS4	TCCTCCGCTTATTGATATGC
*TEF*1	EF1-728F	CATCGAGAAGTTCGAGAAGG
	EF1-986R	TACTTGAAGGAACCCTTACC
*TUB*2	Bt-2a	GGTAACCAAATCGGTGCTGCTTTC
	Bt-2b	ACCCTCAGTGTAGTGACCCTTGGC

**Table 2:** The identities and GenBank accession numbersof *B. dothidea* species used for phylogenetic analyses.

Species	Isolate Accession	Host	Country	GenBank Accession No. (ITS, *TEF1*, *TUB2*)
*Botryosphaeria dothidea*	zdcy-1	*Prunus avium*	China	PV248673, PV250227, PV250231
*B*. *dothidea*	zdcy-2	*Prunus avium*	China	PV248674, PV250228, PV250232
*B*. *dothidea*	zdcy-3	*Prunus avium*	China	PV248675, PV250229, PV250233
*B*. *dothidea*	zdcy-4	*Prunus avium*	China	PV248676, PV250230, PV250234
*B*. *dothidea*	CMW7999	*Ostrya* sp.	Switzerland	AY236948, AY236897 AY236926
*B*. *dothidea*	CMW8000	*Prunus* sp.	Switzerland	AY236949, AY236898 AY236927
*B*. *dothidea*	CMW9075	*Populus nigra*	New Zealand	AY236950, AY236899 AY236928
*B*. *dothidea*	CMW7780	*Fraxinus excelsior*	Switzerland	AY236947, AY236896 AY236925
*B*. *dothidea*	CBS100564	*Paeonia* sp.	The Netherlands	KX464085, KX464555 KX464781
*B*. *ribis*	CMW7772	*Ribes* sp.	USA	AY236935, AY236877 AY236906
*B*. *ribis*	CMW7773	*Ribes* sp.	USA	AY236936, AY236878 AY236907
*B*. *parva*	CMW9077	*Actinidia deliciosa*	New Zealand	AY236939,AY236884 AY236913
*B*. *parva*	CMW9078	*Actinidia deliciosa*	New Zealand	AY236940, AY236885 AY236914
*B*. *parva*	CMW9079	*Actinidia deliciosa*	New Zealand	AY236941, AY236886 AY236915
*B*. *parva*	CMW9080	*Populus nigra*	New Zealand	AY236942, AY236887 AY236916
*B*. *fusispora*	MFLUCC 11-0507	*Caryota* sp.	Thailand	JX646788, JX646853 JX646838
*B*. *fusispora*	MFLUCC 10-0098	*Entada* sp.	Thailand	NR121552, JX646854 JX646839
*B*. *obtusa*	CMW7774	*Ribes* sp.	USA	AY236953, AY236902 AY236931
*B*. *obtusa*	CMW7775	*Ribes* sp.	USA	AY236954, AY236903 AY236932
*B*. *rhodina*	CMW 10130	*Vitex donniana*	Uganda	AY236951, AY236900 AY236929
*B*. *rhodina*	CMW9074	*Pinus* sp.	Mexico	AY236952, AY236901 AY236930
*B*. *corticis*	ATCC 22927	*Vaccinium* sp.	USA	DQ299247, EF614931 EU673108
*B*. *lutea*	CMW9076	*Malus* sp.	New Zealand	AY236946, AY236893 AY236922
*B*. *stevensii*	CMW7060	*Fraxinus excelsior*	Netherlands	AY236955, AY236904 AY236933
*Guignardiaphiloprina*	CMW7063	*Taxus baccata*	Netherlands	AY236956, AY236905 AY236934

CMW: Culture Collection of the Forestry and Agricultural Biotechnology Institute, University of Pretoria; CBS: Culture Collection of the Westerdijk Fungal Biodiversity Institute (formerly CentralbureauvoorSchimmelcultures), Utrecht, the Netherlands; ATCC: American Type Culture Collection; MFLUCC: Mae Fah Luang University Culture Collection, Chiang Rai, Thailand.

## Data Availability

The data presented in this study are available in the manuscript. All sequence data are available in the NCBI database, following the accession numbers in the manuscript.
